# Sesame Extract Promotes Chemopreventive Effect of Hesperidin on Early Phase of Diethylnitrosamine-Initiated Hepatocarcinogenesis in Rats

**DOI:** 10.3390/pharmaceutics13101687

**Published:** 2021-10-14

**Authors:** Napaporn Khuanphram, Sirinya Taya, Prachya Kongtawelert, Rawiwan Wongpoomchai

**Affiliations:** 1 Department of Biochemistry, Faculty of Medicine, Chiang Mai University, Chiang Mai 50200, Thailand; napaporn_khuan@cmu.ac.th; 2 Functional Food Research Unit, Science and Technology Research Institute, Chiang Mai University, Chiang Mai 50200, Thailand; sirinya.t@cmu.ac.th; 3 Thailand Excellence Center for Tissue Engineering and Stem Cells, Department of Biochemistry, Faculty of Medicine, Chiang Mai University, Chiang Mai 50200, Thailand; prachya.k@cmu.ac.th

**Keywords:** hesperidin, sesamin, diethlynitrosamine, cancer chemoprevention, preneoplastic lesion

## Abstract

The combination of natural products is an alternative approach to achieving chemopreventive potential. Accordingly, citrus hesperidin exhibits numerous biological activities, including anticarcinogenic activities, while the sesamin in sesame exhibits potent anticancer activities and lipid-lowering effects. We investigated the cancer chemopreventive effects of mixed sesame and orange seed extract (MSO) containing hesperidin and sesamin in diethylnitrosamine (DEN)-induced hepatocarcinogenesis. Rats were injected with DEN once a week for 3 weeks to induce hepatocarcinogenesis. Rats were fed with MSO and various compositions that included sesame extract (SE) and hesperidin. The 10-week administration of MSO more effectively inhibited the number and size of hepatic GST-P-positive foci than hesperidin in DEN-initiated rats. MSO and hesperidin decreased the number of PCNA-positive hepatocytes but increased the apoptotic cells in DEN-induced rats. Furthermore, MSO and its constituents suppressed hepatic triglyceride content concurrently along with the expression of fatty acid synthase. Although the 5-week administration of MSO or hesperidin did not alter hepatic, preneoplastic lesion formation in DEN-initiated rats, it alleviated DEN-induced hepatotoxicity. MSO and its applied compositions did not impact upon the cytochrome P450 system. In conclusion, sesame extract promoted the chemopreventive effect of hesperidin on DEN-induced early stage of hepatocarcinogenesis in rats. The inhibitory mechanisms are likely involved with the induction of cell apoptosis, suppression of cell proliferation and modulation of hepatic lipogenesis. This study may provide revelations in the development of alternative treatments against hepatocellular carcinoma.

## 1. Introduction

Hepatocellular carcinoma (HCC), the most common type of liver neoplasm, is the leading cause of cancer-related death worldwide. This was particularly true in 2020, when HCC accounted for approximately 905,700 new cases and 810,200 deaths [1]. The main risk factors for HCC not only occur from chronic viral hepatitis infection but also from aflatoxin-contaminated foodstuffs, heavy alcohol intake and obesity [2]. Treatments for liver cancer involve various therapies including surgery, radiation and chemotherapeutic drugs. These treatments and drugs are administered based on the stage of the cancer, liver function and patient performance status [3]. To date, numerous cancer patients have reported to have suffered from the side effects of chemotherapy. These reported side effects include fatigue, nausea, hair loss, anemia and infection [4]. Several preclinical studies have shown that some phytochemicals have exhibited potent antioxidant and anti-inflammatory properties and may provide a suitable alternative strategy in delivering treatment for HCC [5]. Anthocyanin-rich blackcurrant extract possibly inhibits hepatic nodule formation in diethylnitrosamine-initiated rats by modulation of the NF-kB signaling pathway, inhibition of abnormal cell proliferation and induction of apoptosis via the upregulation of Bax and the downregulation of Bcl-2 expression [6,7]. In addition, ethanolic extract of ginger rhizome could suppress the formation of preneoplastic lesions in diethylnitrosamine-initiated and carbon tetrachloride-promoted hepatocarcinogenesis in rats. The ginger extract was also found to decrease various hepatic growth factor levels including VEGF, FGF and TGF-β1 [8]. Furthermore, a treatment of star anise (*Illicium verum*) on the carcinogens of rats could reduce the incidence of liver nodules and could result in lower lipid peroxidation in erythrocytes [9].

Alternative medicine, particularly with regard to the usage of herbal formulas, has long been employed for the alleviation of cancer. The administration of alternative treatments could synergize their chemopreventive potential and reduce the potential toxicity caused by either high doses or the long-term treatment of a single medicinal plant [10,11]. Several studies have extensively investigated the pharmacological effects of herbal medicine formulas or combined herbal extracts on liver cancer development. Combined extracts of *Astragalus membranaceus* and *Salvia miltiorrhiza* presented an inhibitory effect on the progression of HCC by decreasing the expression of preneoplastic lesion markers and by extenuating the degree of fibrosis in diethylnitrosamine-induced rat hepatocarcinogenesis [12,13,14]. Moreover, a Songyou Yin herbal formula containing 5 kinds of medicinal plants delayed HCC formation and inhibited tumor invasion and metastasis in athymic mice models [15]. Furthermore, the decoction of several herbs, including *Panax ginseng*, *Atractylodis macrocephalae* and *Glycyrrhiza uralensis*, reduced tumor recurrence and increased the survival rate in HCC patients by T-lymphocyte cell modulation [16].

Hesperidin, a citrus bioflavonoid comprised of 3,5,7-trihydroxy flavanone-7-rhamnoglucosides exhibited various biological and pharmacological properties including antioxidation [17], anti-inflammation [18] and vascular protection properties [19], as well as lipid-lowering actions [20] and anticarcinogenicity [21,22]. Sesamin, a major furofuran lignan present in sesame, exhibited considerable effects on carbohydrate and lipid metabolism, blood pressure regulation and host defense mechanisms [23]. Furthermore, sesamin could either prevent or delay cancer development via different actions [24,25]. However, there have only been a few reports on the anticarcinogenicity of a combined extract of hesperidin and sesamin. Therefore, to cast light on the combination effect of hesperidin and sesamin on cancer prevention, a diethylnitrosamine-initiated rat model was used to investigate its effects on the early stage of hepatocarcinogenesis in rats and its possible inhibitory mechanisms.

## 2. Materials and Methods

### 2.1. Chemicals

Diethylnitrosamine (DEN), diaminobenzidene (DAB), gallic acid and resorufin were purchased from Sigma Aldrich (St. Louis, MO, USA). The antibodies of rabbit polyclonal GST-placental form (GST-P), rabbit monoclonal fatty acid synthase (FAS) and mouse monoclonal proliferating cell nuclear antigen (PCNA) antibodies were acquired from MBL (Nagoya, Japan), Cell Signaling Technology (Danvers, MA, USA) and BioLegend (San Diego, CA, USA), respectively. Envision^TM^ G/2 Doublestain System with Rabbit/Mouse (DAB+/Permanent Red) was obtained from Dako (Glostrup, Denmark). Avidin–biotin–horseradish peroxidase complex (ABC) kit was acquired from Vector Laboratories (Burlingame, CA, USA). ApopTag peroxidase in situ Apoptosis Detection Kit was obtained from Merck (Kenilworth, NJ, USA). Triglyceride kit assay was received from HUMAN Diagnostics Worldwide (Wiesbaden, Germany). β-Nicotinamide adenine dinucleotide phosphate (NADPH) was supplied by Oriental Yeast Co., Ltd. (Tokyo, Japan).

### 2.2. Test Compounds

Mixed sesame and orange seed extract (MSO), and sesame extract (SE) (lot number JN00101), were provided by EMILY (Samut Prakan, Thailand) Co., Ltd. One gram of MSO consisted of approximately 770, 100, 100 and 20 mg of black sesame powder, *Citrus aurantium* extract, *Citrus sinensis* extract and sesame extract, respectively. Hesperidin (98% purity) from *Citrus aurantium* peel was obtained from Ruiheng Industry Co., Ltd. (Hefei City, China).

### 2.3. Chemical Analysis

Total phenolic contents of MSO and SE were determined using the colorimetric Folin–Ciocalteu method [26]. MSO, SE or standard gallic acid dissolved in methanol was mixed with Folin–Ciocalteu reagent and incubated with sodium carbonate at 45 °C for 15 min. The optical density at a wavelength of 765 nm was read using a spectrophotometer. Total phenolic content was expressed as mg of gallic acid equivalent per gram of the test substance calculated with the use of the calibrating curve of gallic acid.

Quantification of total protein content of MSO and SE was performed by a Bradford assay. The concentration of standard bovine serum albumin (BSA) ranging from 0 to 200 µg/mL was prepared in distilled water. BSA standard and test samples were assessed for their protein concentrations using Coomassie blue Plus assay reagent. The final blue color product was measured for its optical density at a wavelength of 600 nm.

The amounts of sesamin and hesperidin in MSO and SE were analyzed using HPLC separated on a reverse-phase C18 column (ZORBAX Eclipse Plus 5 µm, 250 × 4.6 mm, Agilent Technologies, Santa Clara, CA, USA). Each tested compound was eluted at a flow rate of 1.0 mL/min and the absorbance was detected at a wavelength of 280 nm. Samples and standard sesamin were dissolved with acetonitrile and separated in terms of their soluble portions using a 0.45 μm nylon syringe filter. The mobile phases were identified with acetonitrile as solvent A and distilled water as solvent B. The running condition was 70:30 for both solvents A and B for the first 12 min, followed by a solution of 50:50 of solvents A and B for the last 3 min. To measure the hesperidin content, samples and the standard were extracted with methanol for a period of 5 min. The mobile phases were initiated as 7 parts of 0.1% *O*-phosphoric acid in water and 3 parts of absolute methanol for 10 min. The known anticarcinogenic phenolic acids and flavonoids were analyzed by reverse-phase HPLC as described elsewhere [27]. Ellagic acid, chlorogenic acid, *p*-coumaric acid, ferulic acid, gallic acid, 4-hydroxybenzoic acid, protocatechuic acid, syringic acid and vanillic acid were used as the standard phenolic acids. Apigenin, catechin, epicatechin, luteolin, quercetin and rutin were the reference flavonoids.

### 2.4. Animals

Three-week-old (70 g) and five-week-old (175 g) male Wistar rats were acquired from the Nomura Siam International Co., Ltd. (Bangkok, Thailand) for studying the effect of MSO and its compositions on initiation and promotion stages, respectively. All rats were acclimatized in stainless-steel cages for a period of one week before initiating the experiment. They were fed a pellet diet and tap water ad libitum with temperature control of 25 ± 1 °C under a dark–light cycle throughout the course of the experiment. All protocols were approved of by the Animal Ethical Committee of the Faculty of Medicine, Chiang Mai University (No. 21/2561).

### 2.5. Effect of MSO and Its Compositions on Early Stages of Hepatocarcinogenesis in Rats

**Figure 1:**
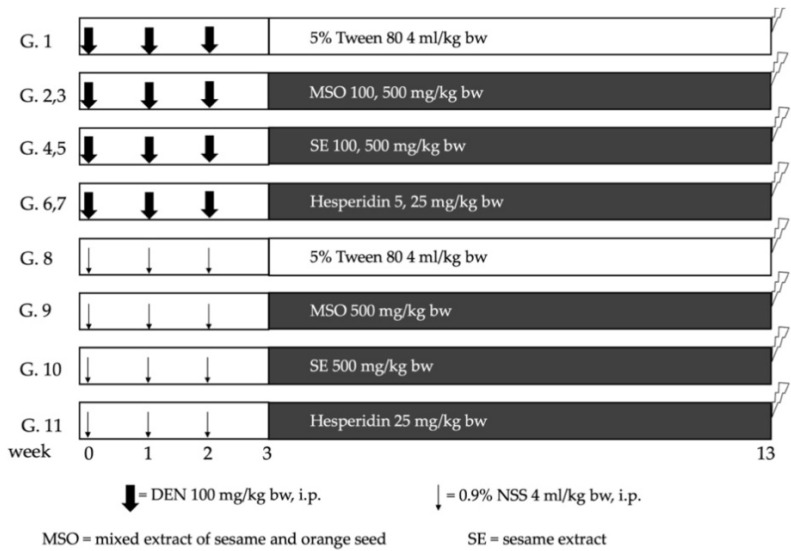
Experimental protocol for studying the effect of MSO and its compositions on the promotion stage of diethylnitrosamine-induced hepatocarcinogenesis in rats. Rats were intraperitoneally injected with diethylnitrosamine (DEN) or normal saline solution (NSS) once a week for 3 weeks. After the last injection, tested compounds were intragastrically administrated 5 days a week for 10 weeks. MSO: mixed sesame and orange seed extract; SE: sesame extract.

MSO at an amount of 100 mg/kg per bw was administered following the recommendations of the Thai FDA. The daily consumption amounted to 1000 mg/day [28]. The amounts of hesperidin and sesame extract given were equivalent to the amount in MSO. The primary objective of this study was to assess the effect of MSO and its compositions on the promotion stage of DEN-initiated hepatocarcinogenesis in six-week-old male rats. According to the study protocol, rats were randomly divided into 11 groups as is shown in Figure 1. During the first 3 weeks of the experiment, Groups 1 to 7 were intraperitoneally injected with 100 mg/kg per bw of diethylnitrosamine (DEN) once a week for 3 weeks, while Groups 8 to 11 were injected with normal saline solution. A week after the last injection, tested compounds were intragastrically administrated 5 days per week for 10 weeks. Groups 1 and 8, which served as positive and negative control groups, respectively, were fed with a 5% Tween-80 vehicle. Low doses of MSO, SE and hesperidin were intragastrically fed to Groups 2, 4 and 6, respectively. Groups 3 and 9 were treated with high doses of MSO, while Groups 5 and 10 received high doses of SE. Lastly, groups 7 and 11 were given high doses of hesperidin. Body weight, along with food and water intake values, were recorded twice a week. At week 13 of the experiment, all rats were euthanized by an overdose of isoflurane inhalation. Subsequently, whole blood was collected from their abdominal veins, while certain vital organs, including the liver, kidneys and spleen, were measured. Three pieces of a liver section were maintained in 10% formalin and the remaining frozen portion was kept in an −80 °C freezer for further immunohistochemistry and molecular analysis, respectively.

To study cancer preventive actions during the initiation stage of MSO and its active ingredients, four-week-old rats were separated into 10 groups. The DEN administration was introduced to Groups 1–5 and a vehicle solution was injected to Groups 6–10 according to the previously stated protocol. The oral administration of the test compounds was initiated 2 weeks before injections were given and feeding continued for 5 weeks. MSO at a low dose was given to Groups 2 and 7, while a high dose was fed to Groups 3 and 8. Low doses of hesperidin were administered to Groups 4 and 9, whereas low doses of hesperidin were fed to Groups 5 and 10. The sacrifice and sampling procedures were similar to those of the previous protocol.

### 2.6. Evaluation of Glutathione S-Transferase Placental form Positive Foci in Liver Tissues

Liver tissue samples from all rats of 5 μm in thickness were deparaffinized and rehydrated with xylene and ethanol, respectively. The specimens were soaked in 3% H_2_O_2_ to inhibit pseudoperoxidase and all specimens were subsequently immersed in 1% skim milk to inactivate nonspecific binding proteins in the tissue. The avidin–biotin–peroxidase complex (ABC) method described by Thumvijit et al. [29] was used to demonstrate GST-P-positive hepatocytes. The liver sections were stained by rabbit polyclonal GST-P antibodies for 2 h at room temperature. Afterwards, they were incubated with biotin-labeled goat antirabbit IgG and subsequently with ABC for 30 min. The sites of peroxidase binding were demonstrated using the DAB method. Sections were counter-stained with hematoxylin for microscopic examination. Number and area of GST-P-positive foci greater than 0.12 mm^2^ and 0.16 mm^2^ were measured over the course of the 5 and 13-week experiments, respectively, using the LAS Interactive Measurement program.

### 2.7. Determination of Cell Proliferation, Apoptosis and Fatty Acid Synthase Expression in Liver Tissues

Liver tissues obtained from the 13-week experiment were assessed to determined cell proliferation, apoptosis and lipogenic enzyme expression using immunohistochemistry. After deparaffinization and rehydration, liver tissues were incubated with proteinase K for 15 min at room temperature before detecting apoptotic hepatocytes using terminal deoxynucleotidyl transferase dUTP nick end labeling (TUNEL) assay. The treated sections were drenched in 3% H_2_O_2_ to quench the endogenous peroxidase and they were then washed with PBS before being incubated with equilibration buffer for 5 min. Afterwards, the working strength of the TdT enzyme was applied to the slides and they were incubated in a humidified chamber at 37 °C for 1 hr. The antidigoxigenin conjugate was incubated for 30 min after adding the stop/wash buffer. The brown color of the TUNEL-positive cells was developed by soaking them in DAB solution for approximately 3 min. Finally, methyl green was used to counterstain the specimens. The number of TUNEL-positive hepatocytes in at least 10 fields of the liver sections were counted under a light microscope.

To measure cell proliferation in preneoplastic lesions and normal areas of the liver tissue, immunostaining of proliferating cell nuclear antigen (PCNA) using the EnVision Doublestain system was performed. After deparaffinization, liver sections were retrieved using citrate buffer at 98 °C for 1 min and H_2_O_2_ for 15 min. Liver slides were then incubated with a dual endogenous enzyme block for 5 min, followed by mouse monoclonal PCNA antibody (1:2000) for 2 h. After samples were incubated with polymer/horseradish peroxidase (HRP) for 10 min, they were drenched with DAB for 30 s and rinsed with distilled water. Next, the double stain block was added and this was followed by the supplementation of rabbit polyclonal GST-P antibody (1:1000) for 1 h before adding the Rabbit/Mouse link. Polymer/alkaline phosphatase was then added and the specimens were subsequently incubated with Permanent Red substrate until the reddish color of the cytoplasmic GST-P staining was observed in the hepatocytes. Numbers of the PCNA-positive hepatocytes labeled in GST-P-positive foci and the surrounding area were counted under a light microscope.

Hepatic lipogenesis was determined using the staining of Fatty Acid Synthase (FASN). The retrieved liver sections were inactivated with nonspecific binding proteins using 1% skim milk for 30 min. After blocking the nonspecific binding proteins, liver sections were treated with normal goat serum and rabbit monoclonal FASN antibody (1:50) overnight. Afterwards, they were incubated with biotin-labeled goat antirabbit IgG using an ABC kit for 30 min. The sites of peroxidase binding were indicated by DAB. Finally, the liver sections were counterstained with hematoxylin and dehydrated with sequential immersing in ethanol and xylene before being slide mounted. The number of FASN positive cells in the liver tissues were counted under a light microscope. 

### 2.8. Determination of Hepatic Triglyceride Content

Frozen liver tissues obtained from the 13-week experiment were homogenized with methanol before chloroform was added and they were allowed to stand in the dark for 16 h in order to extract the lipids. After the soluble chloroform was evaporated, the remaining portion was redissolved in 10% BSA to measure triglyceride content following the manufacturer’s instructions.

### 2.9. Determination of Phases I and Phases II Xenobiotic Metabolizing Enzyme Activities

Frozen liver tissues obtained from 5-week experiments were homogenized using homogenizing buffer that consisted of 1.15% (*w*/*v*) KCI and 0.25 PMSF. The tissue samples were continually centrifuged at 10,000 rpm at 4 °C for 20 min. The obtained supernatant was then collected and specimens were further centrifuged at 100,000 rpm at 4 °C for 60 min. Finally, the cytosolic supernatant and microsomal pellets were evaluated for protein content using the Lowry method and were then maintained at −20 °C and −80 °C, respectively.

Activities of cytochrome P450 (CYP) 1A1, 1A2 and 3A2 were determined according to the method described by Suwannakul et al. [30] using reactions of Ethoxyresorufin-O-demethylation (EROD), methoxyresorufin O-demethylation (MROD) and erythromycin N-demethylation (ENDM), respectively. The microsomal fraction was added into a reaction mixture containing Tris buffer at a pH of 7.8, along with NADPH and EROD for CYP1A1 or MROD for CYP1A2 in a black 96-well plate. The activity of each enzyme was measured using a spectrofluorometer at an excitation wavelength of 520 nm and an emission wavelength of 590 nm. The value was then compared with the standard resorufin curve.

To measure the activity of CYP3A2, either the microsome or standard formaldehyde, along with a reaction mixture containing PBS, 10 mM erythromycin, 150 mM MgC_l2_ and 5 mM NADPH, were incubated at 37 °C for 20 min in a shaking water bath. The enzymatic reaction was stopped by adding trichloroacetic acid. After the resulting supernatant was fractionated by centrifugation at 1900 rpm at 4 °C for 12 min, the color product was developed by reacting with Nash reagent. The enzyme activity was then evaluated using a spectrophotometer at a wavelength of 405 nm with a formaldehyde calibration curve.

The activity of glutathione *S*-transferase (GST) was analyzed using 1-chloro,2,4-dinitrobenzene (CDNB) as a substrate. The cytosolic part was mixed with a reaction mixture containing 10 mM of GSH and 0.2 M of potassium phosphate buffer before the substrate CDNB was added and the specimen was incubated at 37 °C for 20 s. A shift in absorbance at 30 and 90 s was measured at a wavelength of 340 nm, while the GST activity was computed using a molar coefficient of 9.6 M^−1^cm^−1^.

### 2.10. Statistical Analysis

Results were analyzed using Statistical Package for the Social Sciences (SPSS) version 17.0 and represented as values of mean ± SD. One-way ANOVA of variance was applied to test the significance of the different groups followed by the LSD post hoc test. Results were considered significant when *p* was lesser than or equal to 0.05.

## 3. Results

### 3.1. Phytochemical Constituents of Mixed Extract of Sesame and Orange Seed and Sesame Extract

**Figure 2:**
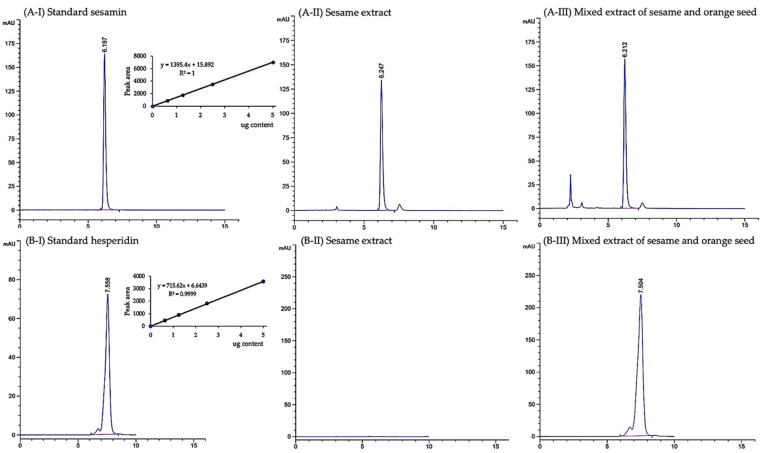
Chromatogram of sesamin (**A**) and hesperidin (**B**) in sesame extract and mixed extract of sesame and orange seed using HPLC.

**Figure 3:**
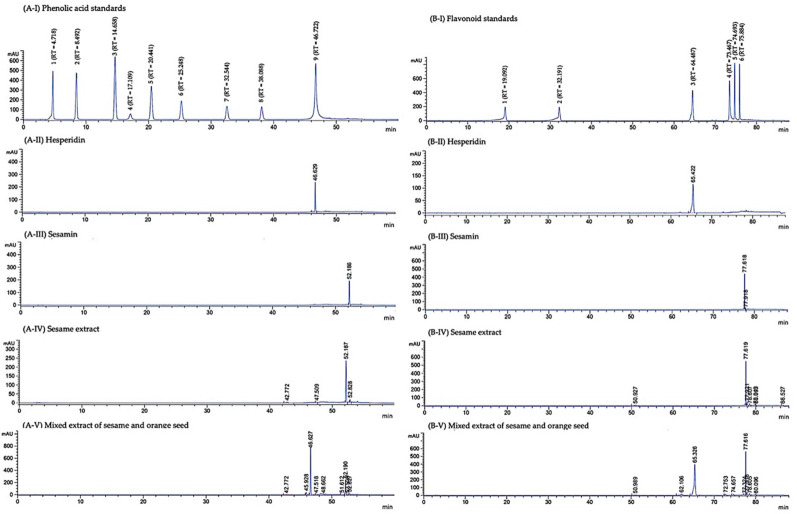
HPLC chromatograms of phenolic acids (**A**) and flavonoids (**B**) of standard hesperidin and sesamin, sesame extract and mixed extract of sesame and orange seed. Gallic acid (1), Protocatechuic acid (2), 4-hydroxybenzoic acid (3), Chlorogenic acid (4), Vanillic acid (5), Syringic acid (6), p-Coumaric acid (7), Ferulic acid (8) and Ellagic acid (9) were used as phenolic acid standards. The standard flavonoids were Catechin (1), Epicatechin (2), Rutin (3), Quercetin (4), Luteolin (5) and Apigenin (6).

One gram of mixed extract of sesame and orange seed (MSO) and sesame extract (SE) contained 57.48 ± 2.09 and 15.79 ± 1.02 mg of phenolic compounds, respectively. Using HPLC analysis, the amounts of sesamin in MSO and SE were 23.37 ± 0.02 and 20.91 ± 0.04 mg/g extract, respectively (Figure 2A). Moreover, 44.16 ± 0.38 mg of hesperidin was found in one gram of MSO (Figure 2B). There are few unknown phenolic acids and flavonoids detected in MSO and SE, however, certain phenolic acids and flavonoids were not found in MSO and SE (Figure 3A,B). The protein and carbohydrate contents in MSO and SE were found to be statistically insignificant. MSO contains 3.31 ± 1.31 mg/g extract of proteins and 125.46 ± 22.50 mg/g extract of carbohydrates, while SE contains 4.11 ± 0.78 mg/g extract of proteins and 119.23 ± 19.24 mg/g extract of carbohydrates.

### 3.2. Effect of MSO and Its Various Compositions on Preneoplastic Lesion Formation in the Livers of Rats

**Figure 4:**
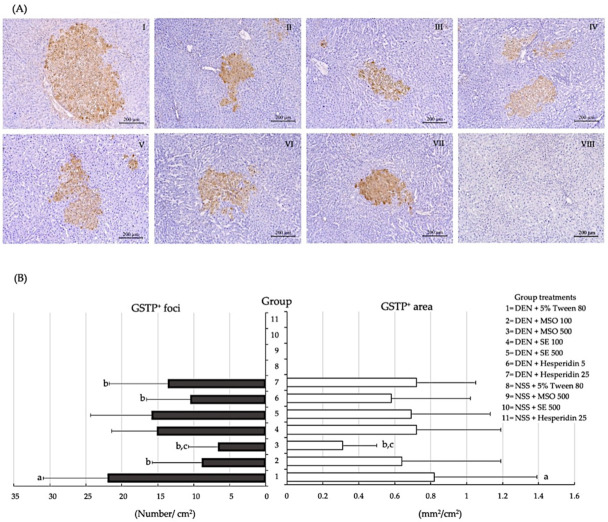
Effect of a 10-week administration of MSO and its compositions on the formation of GST-P-positive foci in rats. (**A**) Characteristics of hepatic GST-P-positive foci, Groups 1–8 are represented as I-VIII, respectively. DEN alone [I], DEN+MSO 100 [II], DEN+MSO 500 [III], DEN+SE 100 [IV], DEN+SE 500 [V], DEN+Hesperidin 5 [VI], DEN+Hesperidin 25 [VII] and a vehicle control [VIII]. (**B**) Number and area of GST-P-positive foci in rats. Values are expressed as mean ± SD. DEN: diethylnitrosamine; GST-P: glutathione *S*-transferase placental form; MSO: mixed sesame and orange seed extract; NSS: normal saline solution; SE: sesame extract. ^a^ significantly different from a negative control group, group 8 (*p* < 0.05). ^b^ significantly different from a positive control group, group 1 (*p* < 0.05). ^c^ significantly different from group 7 (DEN + Hesperidin 25) (*p* < 0.05).

DEN treatment in this study significantly decreased body weight (11.7 ± 3.9% body weight change) but induced hepatic GST-P-positive foci formation in the livers of rats indicating hepatocarcinogenicity (Figure 4). The number and size of GST-P-positive foci induced by DEN were statistically decreased in rats fed with MSO or hesperidin for 10 weeks after DEN initiation, but this reduction was not observed in SE-treated rats. Interestingly, the administration of MSO at high doses exhibited a greater inhibitory effect on preneoplastic lesion development in the livers when compared with the high-dose hesperidin treatment. Neither alteration of body weight nor induction of GST-P-positive foci formation occurred during a 10-week period of administration of MSO and its compositions.

Table 1 presents the results after cessation of DEN treatment for a week, wherein the liver/body weight ratio of rats was decreased. Although the administration of MSO or hesperidin for 5 weeks during DEN-initiation tended to decrease the amounts and areas of GST-P-positive foci in the livers of rats, they could significantly reduce serum ALT levels, indicating a degree of protection against DEN-induced hepatotoxicity (Table 1). 

### 3.3. Effect of MSO and Its Compositions on Cell Proliferation and Apoptosis in the Livers of Rats

**Figure 5:**
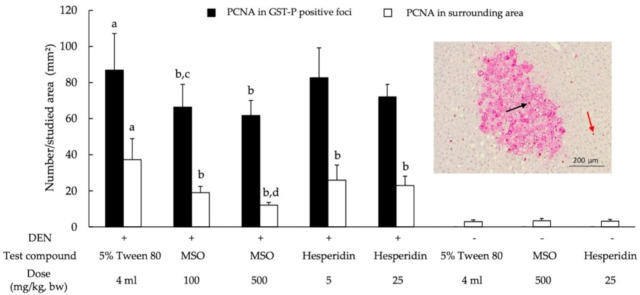
Effect of a 10-week administration of MSO and its compositions on cell proliferation in the livers of rat. Black arrow; PCNA-positive hepatocytes in GST-P-positive foci, red arrow; PCNA-positive hepatocytes in surrounding area. Values are expressed as mean ± SD. DEN: diethylnitrosamine; GST-P: glutathione *S*-transferase placental form; MSO: mixed sesame and orange seed extract; PCNA: proliferating cell nuclear antigen; ^a^ significantly different from a negative control group (*p* < 0.05); ^b^ significantly different from a positive control group (*p* < 0.05); ^c^ significantly different from group 6 (DEN + Hesperidin 5) (*p* < 0.05); ^d^ significantly different from group 7 (DEN + Hesperidin 25) (*p* < 0.05).

PCNA labeling in the nucleus has been used as a biomarker of cell proliferation. Figure 5 depicts the detection of cell proliferation in both preneoplastic lesions and normal areas. The number of PCNA-positive cells in the GST-P-positive foci was higher than in normal tissues (Figure 5). DEN treatment significantly increased the number of PCNA-positive cells when compared with the vehicle control group. Notably, the administration of MSO distinctively reduced the number of PCNA-positive hepatocytes in both preneoplastic lesions and the surrounding liver tissue in DEN-induced rats. Hesperidin reduced PCNA formation only in adjacent GST-P-positive foci. Remarkably, the reduction of cell proliferative markers by the MSO regimen was significantly greater than for the treatment involving hesperidin.

**Figure 6:**
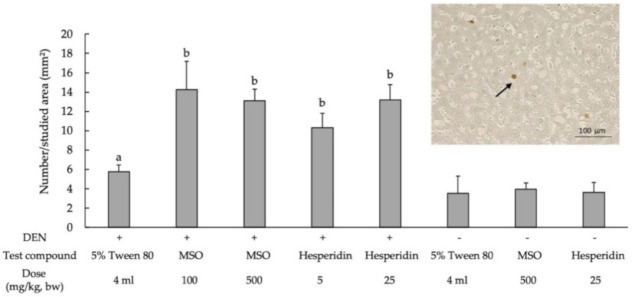
Effect of a 10-week administration of MSO and its compositions on apoptosis in the livers of rat. Black arrow; apoptotic hepatocytes. Values are expressed as mean ± SD. DEN: diethylnitrosamine; GST-P: glutathione *S*-transferase placental form; MSO: mixed sesame and orange seed extract; PCNA: proliferating cell nuclear antigen; ^a^ significantly different from a negative control group (*p* < 0.05); ^b^ significantly different from a positive control group (*p* < 0.05).

The apoptotic hepatocytes in rats were detected by a TUNEL assay (Figure 6). The number of apoptotic cells in the liver tissue samples of DEN-initiated rats was larger than for the nontreated rats (Figure 6). MSO and hesperidin significantly increased the number of apoptotic hepatocytes of DEN-treated rats. Moreover, MSO and its various compositions did not alter liver cell proliferation and apoptosis in rats. The result may suggest that MSO has a selective effect on preneoplastic hepatocytes, implying the safety of MSO under physiological conditions.

### 3.4. Effect of MSO and Its Compositions on Lipid Metabolism in the Livers of Rats

**Figure 7:**
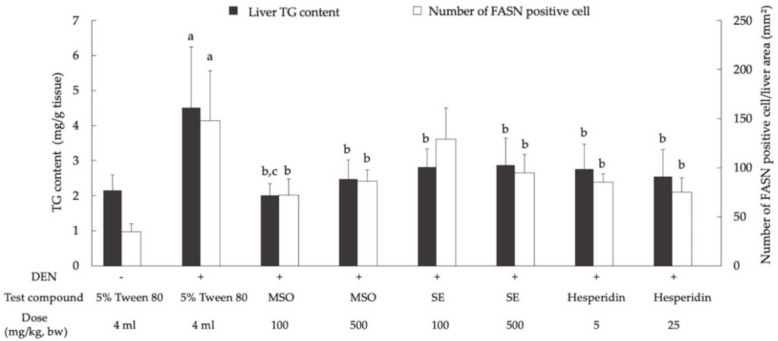
Effect of a 10-week administration of MSO and its compositions on hepatic lipogenesis in the livers of rat. Values are expressed as mean ± SD. DEN: diethylnitrosamine; FASN: fatty acid synthase; MSO: mixed sesame and orange seed extract; SE: sesame extract; TG: triglyceride; ^a^ significantly different from a negative control group (*p* < 0.05); ^b^ significantly different from a positive control group (*p* < 0.05); ^c^ significantly different from group 4 (SE 100) and group 6 (Hesperidin 5) (*p* < 0.05).

DEN-treated groups had significantly increased hepatic triglyceride content and number of FASN-positive cells in liver tissues when compared with the vehicle control group (Figure 7). On the other hand, the administration of MSO and its compositions, including SE and hesperidin, significantly reduced hepatic triglyceride contents and FASN-positive cell numbers in DEN-initiated rats. However, MSO and its various compositions did not affect lipogenesis in rats. 

### 3.5. Effect of MSO and Its Compositions on the Activity of Xenobiotic Metabolizing Enzymes in the Livers of Rats

The administration of MSO and its composition for 5 weeks did not alter the activities of some phase I xenobiotic metabolizing enzymes, including cytochrome P450 (CYP1A1, CYP1A2 and CYP3A2,) as well as the activity of GST detoxifying enzyme in rats (Table 2).

## 4. Discussion

The reduction of HCC incidence is one of the greatest global health challenges we now face. This is due to a significantly high and growing rate of recurrence. Herbal formulas and combined herbal extracts have been traditionally used as alternative ways to treat cancer. Our findings revealed that the combined extracts of sesame extract and hesperidin presented more of a potent chemopreventive effect than its constituents in DEN-initiated hepatocarcinogenesis in rats.

Glutathione *S*-transferase placental form (GST-P) expression in the livers of rats was markedly induced in preneoplastic foci and nodules. It can be detected in a single cell as early as 2–3 days after hepatocarcinogenic induction [31]. DEN is a classical hepatocarcinogen that has been frequently used in experimental carcinogenesis. After DEN is metabolized by hepatic CYP2E1, the resulting alkylating metabolites attack the *N*^7^ atom in the guanine base of nucleic acid, leading to DEN adduct and various gene alterations in the liver [32]. The mutation of oncogenes and tumor suppressor genes can force the dysregulation of biochemical signaling pathways that are associated with cellular proliferation, survival and differentiation [33]. Various studies on the chemopreventive potential of natural products have revealed that several phytochemicals were able to modulate multiple signaling pathways that disturb cell proliferation and cell cycle regulation at each stage of carcinogenesis [34,35]. We found that the administration of hesperidin for 10 weeks after DEN initiation could reduce the number and size of GST-P-positive foci formation in the livers of rats. Furthermore, it increased cell apoptosis but decreased cell proliferation in the liver, particularly in preneoplastic lesions. These results are in accordance with the findings of certain other reports which found that hesperidin could suppress cell proliferation and stimulate cell death in rat hepatocarcinogenesis. Hesperidin modulated exosome production and autophagy, resulting in the suppression of preneoplastic cell proliferation in DEN-initiated rats [36]. It also disrupted the induction of both canonical and noncanonical Wnt pathways in thioacetamide-induced hepatocarcinogenesis in rats [37]. Furthermore, other outcomes have suggested that hesperidin could recover oxidative stress and downregulate the PI3K/Akt pathway in DEN-induced rats [38]. 

Sesame lignans are one type of polyphenolic compounds present in sesame. Sesamin, a major lignan found in sesame extract, has been recognized for its distinct biological activities [39,40]. Sesame extract at the present dosage did not inhibit preneoplastic development in the liver of DEN-initiated rats. Notably, the treatment of the combined extract of sesame extract and hesperidin significantly reduced GST-P-positive foci formation when compared with the treatment of hesperidin alone. Therefore, it might be suggested that sesame extract promotes the inhibitory effect of hesperidin on the early stage of DEN-induced hepatocarcinogenesis. Although the concentrations of sesamin in MSO and SE were slightly different, the total phenolic compound content of MSO was greater than that of SE due to hesperidin content. Therefore, hesperidin was a main phenolic ingredient in MSO. It has been implied that sesamin plays an indirect role in the chemopreventive activity of MSO, while hesperidin and other unknown polyphenols might directly act upon the anticarcinogenicity of MSO.

Hepatic lipid accumulation is determined by the balance between fatty-acid oxidation and fatty-acid biosynthesis [41]. It has been known that the genes involved in fatty-acid biosynthesis were generally upregulated at both the transcriptional and protein stages in a variety of cancers, particularly HCC [42]. In terms of the overexpression of fatty acid synthase (FASN), one critical enzyme in de novo lipogenesis was found more in HCC tissues when compared with nontumorous liver tissues [43,44]. Our study also found an increase in FASN expression in the livers of rats initiated by DEN. This enzyme employs a specific response in the production of palmitate, which is the substrate of the cell membranes, while also involving the palmitoylation of membrane proteins, certain second messenger molecules and the cellular energy supplier used for normal cell survival and cancer cell division [45,46]. Interestingly, the administration of MSO and its compositions significantly reduced triglyceride deposition and FASN expression in the livers of DEN-initiated rats. Consequently, it can be suggested that both hesperidin and sesame extract have functions related to the ablation of lipogenesis during the promotion stage of chemically induced hepatocarcinogenesis.

Recently, the discovery of natural products with anticarcinogenic properties has dramatically escalated. Nonetheless, the efficacy of many of these natural compounds requires a high dose and a long period for administration resulting in a narrow margin of safety [10,11]. A combination of these natural products has been suggested as an alternative way to increase their chemopreventive potential, particularly in the treatment of liver cancer which is known to be highly resistant to chemotherapies. Our results found that a regimen of combined extracts of hesperidin with sesame extract did improve anticarcinogenicity against DEN-induced early stage hepatocarcinogenesis in rats when compared with the hesperidin treatment alone. Whilst hesperidin, but not sesame extract, in MSO exhibited cancer chemopreventive efficacy, both of them could reduce lipid accumulation by suppression of FASN expression. Therefore, it has been suggested that sesame extract could enhance the chemopreventive efficiency of hesperidin, resulting in the alleviation of preneoplastic lesion development in the livers of DEN-initiated rats.

Remarkably, neither hesperidin nor MSO inhibited preneoplastic lesion formation in the livers of rats during the initiation stage. The attenuated acute hepatotoxicity of DEN initiation was indicated by the reduction of serum ALT levels in rats. This might support a protective effect of MSO at the onset of hepatocarcinogenesis. Though a combination of plant extracts could gain an advantage as an alternative remedy, there is a risk of either drug–herb or herb–herb interactions, particularly those mediated by xenobiotic metabolizing systems [47,48]. This study determined that the 5-week administration of MSO or hesperidin did not alter the activities of some important cytochrome P450 isozymes and detoxifying enzymes in rats. It was therefore suggested that MSO might be safe when consumed with food or drugs due to a low risk for any potential interaction with other drugs and herbal extracts.

## 5. Conclusions

Mixed sesame and orange seed extract (MSO) can exert a chemopreventive effect at the promotion stage through the inhibition of cell proliferation and the enhancement of apoptosis, as well as via the suppression of hepatic de novo lipogenesis and a protective effect on the acute toxic phase during the initiation stage of diethylnitrosamine-induced hepatocarcinogenesis in rats. Hesperidin is an anticarcinogenic ingredient in MSO, and sesame extract did exhibit cancer chemopreventive potential. This combined extract can contribute to a supportive approach in the elevation of malignant tumor prevention efficiency.

## Figures and Tables

**Table 1:** Effect of a 5-week administration of MSO and its compositions on liver weight, serum ALT level and GST-P-positive foci formation in rats.

Group	Treatment (mg/kg bw)	Final Body Weight (g)	Liver Weight (g)	Liver/Body Weight Ratio	GST-P Number/ Liver Area (cm^2^)	GST-P Area(mm^2^)/ Liver Area (cm^2^)	ALT (U/L)
1	DEN + 5% Tween 80	277.2 ± 16.0	7.86 ± 0.72 ^a^	2.84 ± 0.25 ^a^	12.6 ± 3.5 ^a^	0.56 ± 0.43 ^a^	60.1 ± 13.7 ^a^
2	DEN + MSO 100	262.2 ± 27.1	7.73 ± 1.07	2.96 ± 0.35	13.2 ± 4.4 ^a^	0.31 ± 0.43 ^a^	50.8 ± 10.5 ^b^
3	DEN + MSO 500	245.7 ± 20.5 ^b^	7.23 ± 0.70	2.95 ± 0.20	14.1 ± 6.1 ^a^	0.53 ± 0.45 ^a^	53.0 ± 10.5 ^b^
4	DEN + Hesperidin 5	252.5 ± 43.2	6.67 ± 2.05	2.74 ± 0.78	9.2 ± 4.4 ^a^	0.30 ± 0.32 ^a^	43.3 ± 9.3 ^b^
5	DEN + Hesperidin 25	263.9 ± 22.0	7.49 ± 0.67	2.84 ± 0.19	14.7 ± 4.4 ^a^	0.23 ± 0.22 ^a^	43.2 ± 9.1 ^b^
6	NSS + 5% Tween 80	289.0 ± 19.8	10.19 ± 0.92	3.52 ± 0.14	0	0	33.0 ± 2.7
7	NSS + MSO 100	296.0 ± 39.9	11.31 ± 1.74	3.82 ± 0.26	0	0	29.0 ± 2.6
8	NSS + MSO 500	279.0 ± 40.4	10.11 ± 0.96	3.65 ± 0.24	0	0	30.0 ± 2.4
9	NSS + Hesperidin 5	291.0 ± 42.2	10.45 ± 1.96	3.58 ± 0.27	0	0	31.6 ± 3.7
10	NSS + Hesperidin 25	284.0 ± 25.1	10.85 ± 0.78	3.83 ± 0.27	0	0	32.4 ± 3.6

Values are expressed as mean ± SD. ALT: alanine aminotransferase; DEN: diethylnitrosamine; GST-P: glutathione *S*-transferase placental form; MSO: mixed sesame and orange seed extract; NSS: normal saline solution; SE: sesame extract. ^a^ significantly different from Group 6 (*p* < 0.05), ^b^ significantly different from Group 1 (*p* < 0.05).

**Table 2:** Effect of the 5-week administration of MSO and active ingredients on xenobiotic metabolizing enzyme activities in the livers of rats.

Group	Chemical	Treatment (mg/kg, bw)	Enzyme Activities			
			CYP1A1 ^a^	CYP1A2 ^a^	CYP3A2 ^b^	GST ^c^
6	NSS	5% Tween 80	4.06 ± 1.09	2.34 ± 0.63	6.96 ± 1.33	48.5 ± 7.49
7	NSS	MSO 100	6.48 ± 2.26	2.63 ± 1.21	7.01 ± 0.42	45.6 ± 4.24
8	NSS	MSO 500	4.12 ± 1.93	1.61 ± 0.43	6.21 ± 0.93	52.6 ± 4.49
9	NSS	Hesperidin 5	5.67 ± 1.51	2.68 ± 0.58	7.47 ± 1.57	52.5 ± 7.49
10	NSS	Hesperidin 25	6.13 ± 1.40	2.70 ± 0.73	7.59 ± 1.15	51.6 ± 4.54

Values are expressed as mean ± SD. ^a^ = fmole/min/mg protein; ^b^ = pmole/min/mg protein; ^c^ = ×10^−2^ unit/mg protein; CYP: cytochrome P450; GST: glutathione *S*-transferase; MSO: mixed sesame and orange seed extract; NSS: normal saline solution.

## Data Availability

Not applicable.

## References

[1] Sung H., Ferlay J., Siegel R.L., Laversanne M., Soerjomataram I., Jemal A., Bray F. (2021). Global cancer statistics 2020: GLOBOCAN estimates of incidence and mortality worldwide for 36 cancers in 185 countries. CA Cancer J. Clin..

[2] Llovet J.M., Zucman-Rossi J., Pikarsky E., Sangro B., Schwartz M., Sherman M., Gores G. (2016). Hepatocellular carcinoma. Nat. Rev. Dis. Primers.

[3] Raza A., Sood G.K. (2014). Hepatocellular carcinoma review: Current treatment, and evidence-based medicine. World J. Gastroenterol..

[4] Carr C., Ng J., Wigmore T. (2008). The side effects of chemotherapeutic agents. Curr. Anaesth. Crit. Care.

[5] Darvesh A.S., Aggarwal B.B., Bishayee A. (2012). Curcumin and liver cancer: A review. Curr. Pharm. Biotechnol..

[6] Bishayee A., Mbimba T., Thoppil R.J., Haznagy-Radnai E., Sipos P., Darvesh A.S., Folkesson H.G., Hohmann J. (2011). Anthocyanin-rich black currant (*Ribes nigrum* L.) extract affords chemoprevention against diethylnitrosamine-induced hepatocellular carcinogenesis in rats. J. Nutr. Biochem..

[7] Bishayee A., Thoppil R.J., Mandal A., Darvesh A.S., Ohanyan V., Meszaros J.G., Haznagy-Radnai E., Hohmann J., Bhatia D. (2013). Black currant phytoconstituents exert chemoprevention of diethylnitrosamine-initiated hepatocarcinogenesis by suppression of the inflammatory response. Mol. Carcinog..

[8] Mansour M.A., Bekheet S.A., Al-Rejaie S.S., Al-Shabanah O.A., Al-Howiriny T.A., Al-Rikabi A.C., Abdo A.A. (2010). Ginger ingredients inhibit the development of diethylnitrosoamine induced premalignant phenotype in rat chemical hepatocarcinogenesis model. Biofactors.

[9] Yadav A.S., Bhatnagar D. (2007). Chemo-preventive effect of Star anise in N-nitrosodiethylamine initiated and phenobarbital promoted hepato-carcinogenesis. Chem. Biol. Interact..

[10] Phua D.H., Zosel A., Heard K. (2009). Dietary supplements and herbal medicine toxicities-when to anticipate them and how to manage them. Int. J. Emerg. Med..

[11] Teo D.C., Ng P.S., Tan S.H., Lim A.T., Toh D.S., Chan S.Y., Cheong H.H. (2016). Drug-induced liver injury associated with complementary and alternative medicine: A review of adverse event reports in an Asian community from 2009 to 2014. BMC Complement. Altern. Med..

[12] Rui W., Xie L., Liu X., He S., Wu C., Zhang X., Zhang L., Yang Y. (2014). Compound *Astragalus* and *Salvia miltiorrhiza* extract suppresses hepatocellular carcinoma progression by inhibiting fibrosis and PAI-1 mRNA transcription. J. Ethnopharmacol..

[13] Boye A., Wu C., Jiang Y., Wang J., Wu J., Yang X., Yang Y. (2015). Compound *Astragalus* and *Salvia miltiorrhiza* extracts modulate MAPK-regulated TGF-beta/Smad signaling in hepatocellular carcinoma by multi-target mechanism. J. Ethnopharmacol..

[14] Wu C., Kan H., Hu M., Liu X., Boye A., Jiang Y., Wu J., Wang J., Yang X., Yang Y. (2018). Compound *Astragalus* and *Salvia miltiorrhiza* extract inhibits hepatocarcinogenesis via modulating TGF-beta/TbetaR and Imp7/8. Exp. Ther. Med..

[15] Zhang Q.-B., Meng X.-T., Jia Q.-A., Bu Y., Ren Z.-G., Zhang B.-H., Tang Z.-Y. (2016). Herbal compound Songyou Yin and moderate swimming suppress growth and metastasis of liver cancer by enhancing immune function. Integr. Cancer Ther..

[16] Chen F., Zhong Z., Tan H.Y., Guo W., Zhang C., Tan C.-w., Li S., Wang N., Feng Y. (2020). Uncovering the anticancer mechanisms of Chinese herbal medicine formulas: Therapeutic alternatives for liver cancer. Front. Pharmacol..

[17] Pari L., Karthikeyan A., Karthika P., Rathinam A. (2015). Protective effects of hesperidin on oxidative stress, dyslipidaemia and histological changes in iron-induced hepatic and renal toxicity in rats. Toxicol. Rep..

[18] Li R., Li J., Cai L., Hu C.M., Zhang L. (2008). Suppression of adjuvant arthritis by hesperidin in rats and its mechanisms. J. Pharm. Pharmacol..

[19] Morand C., Dubray C., Milenkovic D., Lioger D., Martin J.F., Scalbert A., Mazur A. (2011). Hesperidin contributes to the vascular protective effects of orange juice: A randomized crossover study in healthy volunteers. Am. J. Clin. Nutr..

[20] Roza J.M., Xian-Liu Z., Guthrie N. (2007). Effect of citrus flavonoids and tocotrienols on serum cholesterol levels in hypercholesterolemic subjects. Altern. Ther. Health Med..

[21] Kamaraj S., Ramakrishnan G., Anandakumar P., Jagan S., Devaki T. (2009). Antioxidant and anticancer efficacy of hesperidin in benzo(a)pyrene induced lung carcinogenesis in mice. Investig. New Drugs.

[22] Kongtawelert P., Wudtiwai B., Shwe T.H., Pothacharoen P., Phitak T. (2020). Inhibitory effect of hesperidin on the expression of programmed death ligand (PD-L1) in breast cancer. Molecules.

[23] Dar A.A., Arumugam N. (2013). Lignans of sesame: Purification methods, biological activities and biosynthesis—A review. Bioorg. Chem..

[24] Harikumar K.B., Sung B., Tharakan S.T., Pandey M.K., Joy B., Guha S., Krishnan S., Aggarwal B.B. (2010). Sesamin manifests chemopreventive effects through the suppression of NF-kappa B-regulated cell survival, proliferation, invasion, and angiogenic gene products. Mol. Cancer Res..

[25] Stewart B.W., Bray F., Forman D., Ohgaki H., Straif K., Ullrich A., Wild C.P. (2016). Cancer prevention as part of precision medicine: ‘plenty to be done’. Carcinogenesis.

[26] Wolfe K., Wu X., Liu R.H. (2003). Antioxidant activity of apple peels. J. Agric. Food Chem..

[27] Chariyakornkul A., Punvittayagul C., Taya S., Wongpoomchai R. (2019). Inhibitory effect of purple rice husk extract on AFB1-induced micronucleus formation in rat liver through modulation of xenobiotic metabolizing enzymes. BMC Complement. Altern. Med..

[28] Reagan-Shaw S., Nihal M., Ahmad N. (2008). Dose translation from animal to human studies revisited. FASEB J..

[29] Thumvijit T., Taya S., Punvittayagul C., Peerapornpisal Y., Wongpoomchai R. (2014). Cancer chemopreventive effect of *Spirogyra neglecta* (Hassall) Kützing on diethylnitrosamine-induced hepatocarcinogenesis in rats. Asian Pac. J. Cancer Prev..

[30] Suwannakul N., Punvittayagul C., Jarukamjorn K., Wongpoomchai R. (2015). Purple rice bran extract attenuates the aflatoxin B1-induced initiation stage of hepatocarcinogenesis by alteration of xenobiotic metabolizing enzymes. Asian Pac. J. Cancer Prev..

[31] Muramatsu M., Sakai M. (2006). Mechanisms of a tumor marker, glutathione transferase P, expression during hepatocarcinogenesis of the rat. Proc. Jpn. Acad. Ser. B Phys. Biol. Sci..

[32] Gao J., Wang G.-J., Wang Z., Gao N., Li J., Zhang Y.-F., Zhou J., Zhang H.-X., Wen Q., Jin H. (2017). High CYP2E1 activity correlates with hepatofibrogenesis induced by nitrosamines. Oncotarget.

[33] Sever R., Brugge J.S. (2015). Signal transduction in cancer. Cold Spring Harb. Perspect. Med..

[34] Howes M.J., Simmonds M.S. (2014). The role of phytochemicals as micronutrients in health and disease. Curr. Opin. Clin. Nutr. Metab. Care.

[35] Kotecha R.T., Takami A., Espinoza J.L. (2016). Dietary phytochemicals and cancer chemoprevention: A review of the clinical evidence. Oncotarget.

[36] Hasanin A.H., Matboli M., Seleem H.S. (2020). Hesperidin suppressed hepatic precancerous lesions via modulation of exophagy in rats. J. Cell Biochem..

[37] Zaghloul R.A., Elsherbiny N.M., Kenawy H.I., El-Karef A., Eissa L.A., El-Shishtawy M.M. (2017). Hepatoprotective effect of hesperidin in hepatocellular carcinoma: Involvement of Wnt signaling pathways. Life Sci..

[38] Mo’men Y.S., Hussein R.M., Kandeil M.A. (2019). Involvement of PI3K/Akt pathway in the protective effect of hesperidin against a chemically induced liver cancer in rats. J. Biochem. Mol. Toxicol..

[39] Wu M.S., Aquino L.B.B., Barbaza M.Y.U., Hsieh C.L., Castro-Cruz K.A., Yang L.L., Tsai P.W. (2019). Anti-inflammatory and anticancer properties of bioactive compounds from *Sesamum indicum* L.—A review. Molecules.

[40] Andargie M., Vinas M., Rathgeb A., Möller E., Karlovsky P. (2021). Lignans of sesame (*Sesamum indicum* L.): A comprehensive review. Molecules.

[41] Ipsen D.H., Lykkesfeldt J., Tveden-Nyborg P. (2018). Molecular mechanisms of hepatic lipid accumulation in non-alcoholic fatty liver disease. Cell Mol. Life Sci..

[42] Berndt N., Eckstein J., Heucke N., Gajowski R., Stockmann M., Meierhofer D., Holzhutter H.G. (2019). Characterization of lipid and lipid droplet metabolism in human HCC. Cells.

[43] Che L., Pilo M.G., Cigliano A., Latte G., Simile M.M., Ribback S., Dombrowski F., Evert M., Chen X., Calvisi D.F. (2017). Oncogene dependent requirement of fatty acid synthase in hepatocellular carcinoma. Cell Cycle.

[44] Che L., Chi W., Qiao Y., Zhang J., Song X., Liu Y., Li L., Jia J., Pilo M.G., Wang J. (2020). Cholesterol biosynthesis supports the growth of hepatocarcinoma lesions depleted of fatty acid synthase in mice and humans. Gut.

[45] Peck B., Schulze A. (2016). Lipid desaturation—The next step in targeting lipogenesis in cancer?. Febs J..

[46] Röhrig F., Schulze A. (2016). The multifaceted roles of fatty acid synthesis in cancer. Nat. Rev. Cancer.

[47] Wanwimolruk S., Prachayasittikul V. (2014). Cytochrome P450 enzyme mediated herbal drug interactions (Part 1). EXCLI J..

[48] Brewer C.T., Chen T. (2017). Hepatotoxicity of herbal supplements mediated by modulation of cytochrome P450. Int. J. Mol. Sci..

